# On the Use of Scarifying and Cupping

**Published:** 1803-11-01

**Authors:** N. Broussonet


### *On the* USE *of* SCARIFYING *and* CUPPING; *by* N. BROUSSONET, M.D. *Communicated by our Correspondent at Paris.*

WHY have certain remedies, commonly employed by the ancients, been neglected by the moderns, so as hardly to find a trace of them in the works of the present day? Has experience proved that these remedies were altogether useless? or have others, more efficacious, been substituted? No; but it will be answered, they are no longer the fashion.

The author tells us, that he hardly commenced practice when he determined to give a trial to the actual cautery, from which he experienced as surprising advantages as are set forth by authors; in short, such as to justify all that Pouteau has said in its praise. It remained to make trial of cupping and scarifications, but the prejudices of people against this remedy prevented the author from proposing it in private practice. In the army, however, he had ample opportunity of giving this practice a fair trial during three years. Here he found the superiority of scarification in some cases in which blisters and rubefacients are recommended. He confirmed the observations of Stoll, on the advantage of cupping in catarrhal peripneumony, particularly in those cases which prevailed in the army of the western Pyrennees during the winter of 1794. The circumstances in which M. Broussonet was placed, gave few or no opportunities of ascertaining its effects, except in internal diseases; but last year a case occurred to him, which in many points of view the author thinks important.

On the 16th of July, a carter, aged forty, of a bilious temperament, received a kick of a horse under the left hypochondrium; the stroke was so violent as to knock him down senseless; on coming to himself he vomited. When the author arrived, the patient complained loudly of pain, which he felt above the place where the stroke was received; there was no echymosis, the pulse was small and intermitting, and the body covered with a cold sweat; he had taken a little wine, and in this state he continued till evening. During the Author's absence he fell into a state of coma, which returned from time to time, and during which interval he complained much of pain; in this state he found him at six o'clock in the evening, when the part was scarified and cupped. The operation was hardly begun when the patient fell again into coma; the pulse becoming much stronger stronger the cupping was repeated four times, after which the scarified part was covered with a bread and milk poultice, and the section of the abdomen was covered with cloths rung out of emollient decoctions; the patient rested by intervals, and towards the morning he passed with his urine a considerable quantity of blood; this confirmed the author that the spleen and kidney had been violently contused, besides which the small hard pulse, the difficulty of breathing, and the pain which the patient referred to the xiphoid cartilage, left little room to doubt of an injury being done to the diaphragm.

17th. In the morning the scarifications and cupping were repeated. The patient was relieved, the pulse was stronger; the tongue became foul on the right side. Although there was more reason to apprehend a bilious than an inflammatory fever, yet by way of precaution, and to remove spasm, about two ounces of blood were taken from the left arm; the cupping was repeated, and the patient passed a somewhat better night. Since the accident, he had taken but a light tisanne; clysters were frequently administered.

18th. The pain was less violent, the patient was twice cupped, and the urine was as in a state of health.

19th. He found himself much better, and felt an appetite for food, which he was not however permitted to use. The pain was yet considerable on changing position. The author feared a deposition, in which he was justified by the event.

23d. In the morning the patient complained of weakness, and immediately afterwards was in the greatest agonies. When the author arrived, the pulse was not perceivable, the extremities were cold, the teeth were covered with the *fatal* mucus; in short, the patient was about to expire. The author did not long hesitate on the means to be pursued; it was the seventh day from that of the accident; convinced that the animal powers were not exhausted, he still hoped; Two blisters were put on the legs, two on the hypochondria, and cupping glasses were once more applied over deep scarifications on the affected part. Towards noon the patient seemed to return to life; in the evening he was better, and during the night he evacuated a quantity of black bilious matter by stool, which formed probably the deposition, and the sudden evacuation of which into the intestines terminated the disease. Since this day the patient recovered, and in the course of ten days could resume his usual occupation.

From

From this case, the author thinks we may estimate the benefits arising from the application of cupping and scarifying; it enables us to judge too of the excellence of that leading precept, of bleeding repeatedly in contusions of the viscera. The author thinks, that if he had employed this method, that a bilious fever would have been the consequence, and which he could not have checked, or else the system would have been so exhausted as to be unable to make that salutary and critical effort which terminated happily the disease. The progress of the complaint also shews, that when Nature is not disturbed in her operations by an unnecessary and ignorant interference on the part of the practitioner, that she follows in external diseases the same course as in internal ones. In this case, the fourth day of the disease indicated the crisis which took place on the seventh.

